# The association analysis between HLA-A*26 and Behçet’s disease

**DOI:** 10.1038/s41598-019-40824-y

**Published:** 2019-03-14

**Authors:** Jutaro Nakamura, Akira Meguro, Genji Ishii, Takahiro Mihara, Masaki Takeuchi, Yuki Mizuki, Kentaro Yuda, Takahiro Yamane, Tatsukata Kawagoe, Masao Ota, Nobuhisa Mizuki

**Affiliations:** 1 Department of Ophthalmology and Visual Science, Yokohama City University Graduate School of Medicine, Yokohama, Japan; 2 Department of Ophthalmology, Heisei Yokohama Hospital, Yokohama, Japan; 3 Product Strategy Department, PFU Limited, a Fujitsu Company, Yokohama, Japan; 4 Department of Anesthesiology and Critical Care Medicine, Yokohama City University Graduate School of Medicine, Yokohama, Japan; 5 Department of Medicine, Division of Gastroenterology and Hepatology, Shinshu University School of Medicine, Matsumoto, Japan

## Abstract

The strongest genetic risk factor of Behçet’s disease (BD) is HLA-B*51. Our group previously reported that HLA-A*26 is independently associated with the risk of the onset of BD apart from HLA-B*51. Here, we re-evaluated the association between HLA-A*26 and BD in the Japanese population. We also performed a comprehensive literature search and meta-analyzed the extracted published data concerning the relationship between HLA-A*26 and BD to estimate the odds ratio (OR) of HLA-A*26 to BD. In this study, we genotyped 611 Japanese BD patients and 2,955 unrelated ethnically matched healthy controls. Genotyping results showed that the phenotype frequency of HLA-A*26 was higher in BD patients than in controls (OR = 2.12, 95% CI: 1.75–2.56). Furthermore, within the HLA-B*51-negative populations, the phenotype frequency of HLA-A*26 was significantly higher in BD patients than in controls (OR = 3.10, 95% CI: 2.43–3.95). Results obtained from meta-analysis combined with our data showed that the modified OR of HLA-A*26 became 1.80 (95% CI:1.58–2.06), whereas within the HLA-B*51-negative population, the modified OR became 4.02 (95% CI: 2.29–7.05). A subgroup analysis arranged by the geographical regions showed HLA-A*26 is in fact associated with the onset of BD in Northeast Asia (OR = 2.11, 95% CI: 1.75–2.56), but not in the Middle East or in Europe.

## Introduction

Behçet’s disease (BD) is a recurrent multisystem inflammatory disorder characterized by four classical major symptoms consisting of recurrent aphthous oral ulcers, genital ulcers, ocular uveitis, and Erythema-nodosum-like skin lesions. Occasionally the inflammation of BD occurs in tissues and organs throughout the body including the vascular system, the central nervous system, the gastrointestinal tract, the lungs, the kidneys, and various joints. Despite being worldwide, the distribution of BD is higher in an area along the old Silk Route that extends from far Eastern Asia to the Mediterranean Basin^1,2^.

The strongest genetic risk factor of the BD is HLA-B*51. The odds ratio (OR) of HLA-B*51 to the BD was 5.78 with 95% confidential Interval (CI) = 5.00–6.67^3^. The susceptible association of HLA-A*26 to the BD was initially reported from Taiwan, followed by various countries and ethnicities^4–11^. We previously reported that HLA-A*26 was independently and significantly associated with the risk of the onset of BD apart from HLA-B*51^12^. Several studies have reported that HLA-A*26:01 plays a predominant role in causing intense eye inflammations, which lead to uveitis and visual dysfunction, particularly in the Northeast Asian population^4,8^. The etiology of HLA-A*26 related BD causing more frequent and intense inflammations in uveal tissues in these populations is still unknown. Because HLA-A*26, as a risk allele of BD, is independent from HLA-B*51, HLA-A*26 mediated genetic pathways might be different from that of HLA-B*51 in how they develop inflammations. Further investigations to clarify the genetic involvement of HLA-A*26 and its correlation with the particular clinical manifestations of BD phenotypes in the Northeast Asian population might lead to one of the clues to understanding why there are regional disparities of phenotypes within BD patients.

In this study, we investigated the relationship between HLA-A*26 and BD by genotyping 611 Japanese BD patients and 2,955 unrelated ethnically matched healthy controls. In addition to our currently obtained genotyping data, we performed a comprehensive literature search and meta-analyzed the extracted published data concerning the relationship between HLA-A*26 and BD to estimate the synthesized OR of HLA-A*26 to BD. Additionally, we investigated the distribution of HLA-A*26 in the world population and summarized the regional and ethnical disparities of HLA-A*26 involvement in BD patients.

## Results

### HLA-A*26 genotyping

Allele and phenotype frequencies of HLA-A*26 are shown in Table 1. Both allele and phenotype frequencies of HLA-A*26 were significantly higher in the patient group as compared to the healthy controls. Allele frequency: 18.43% in BD vs. 10.93% in controls (OR = 1.84, 95% CI: 1.56–2.18). Phenotype frequency: 35.55% in BD vs. 20.68% in controls (OR = 2.12, 95% CI: 1.75–2.56). In addition to HLA-A*26 allele, -A*11, -A*31, -A*33 were also statistically significant, and the frequencies in the patient group and controls indicated that the genetic association of -A*31 as risk and -A*11, -A*33 as protective type.

**Table 1:** Left: Allele frequencies of HLA-A antigens in the Japanese Behçet’s disease patient population.

HLA-A allele	BD cases (N = 611)		Controls (N = 2,955)		*p*	*Pc*	HLA-A*26 phenotype	BD cases (N = 611)		Controls (N = 2,955)	
	n	Allele freq.	n	Allele freq.				N	Phenotype freq.	N	Phenotype freq.
A*01	17	1.40%	40	0.68%	0.0108	0.1188	A*26 positive	214	35.55%	601	20.68%
A*02	267	22.07%	1,380	23.60%	NS		A*26 negative	388	64.45%	2,305	79.32%
A*03	1	0.08%	25	0.43%	NS		Total	602	100.00%	2,906	100.00%
A*11	75	6.20%	543	9.29%	0.0005	0.0055	Undetermined	9	—	49	—
A*24	434	35.87%	2,225	38.05%	NS						
A*26	223	18.43%	639	10.93%	<0.0001	<0.0001					
A*30	0	0.00%	15	0.26%	NS						
A*31	136	11.24%	498	8.52%	0.0026	0.0286					
A*32	0	0.00%	2	0.03%	NS						
A*33	57	4.71%	481	8.23%	<0.0001	<0.0001					
Total	1,210	100.00%	5,848	100.00%							
Undetermined	12	—	62	—							

Right: Phenotype frequencies of HLA-A*26 in the Japanese Behçet’s disease patient population. *Pc*: *p* value corrected by Bonferroni method; NS: not significant.

### HLA-A*26 frequency within the HLA-B*51-negative population

In the current genotyping study, 314 BD patients (51%) and 2,433 controls (82%) did not carry the HLA-B*51 antigen. The allele and phenotype frequencies of HLA-A*26 within the HLA-B*51-negative populations are shown in Table 2. Both allele and phenotype frequencies of HLA-A*26 were significantly higher in the BD group as compared to the controls. Allele frequency: 23.57% in BD vs. 11.16% in controls (OR = 2.48, 95% CI: 2.02–3.04). Phenotype frequency: 45.22% in BD vs. 21.05% in controls (OR = 3.10, 95% CI: 2.43–3.95). Besides HLA-A*26, -A*33 were statistically significant, and the frequency in the patient group and controls indicated HLA-A*33 as protective type.

**Table 2:** Left: Allele frequencies of the HLA-A antigens in the Japanese Behçet’s disease patients within HLA-B*51 non-carriers.

HLA-A allele	BD cases (N = 314)		Controls (N = 2,433)		*p*	*Pc*	HLA-A*26 phenotype	BD cases (N = 314)		Controls (N = 2,433)	
	N	Allele Freq.	N	Allele Freq.				N	Phenotype freq.	N	Phenotype freq.
A*01	11	1.75%	34	0.71%	0.0064	0.0704	A*26 positive	142	45.22%	504	21.05%
A*02	143	22.77%	1,158	24.03%	NS		A*26 negative	172	54.78%	1,890	78.95%
A*03	1	0.16%	23	0.48%	NS		Total	314	100.00%	2,394	100.00%
A*11	49	7.80%	455	9.44%	NS		Undetermined	0	—	39	—
A*24	219	34.87%	1,880	39.01%	0.045	0.495					
A*26	148	23.57%	538	11.16%	<0.0001	<0.0001					
A*30	0	0.00%	14	0.29%	NS						
A*31	28	4.46%	285	5.91%	NS						
A*32	0	0.00%	2	0.04%	NS						
A*33	29	4.62%	430	8.92%	0.0003	0.0033					
Total	628	100.00%	4,819	100.00%							
Undetermined	0	—	47	—							

Right: Phenotype frequencies of the HLA-A*26 in the Japanese Behçet’s disease patients within HLA-B*51 non-carriers. *Pc*: *p* value corrected by Bonferroni method; NS: not significant.

### Retrieved HLA-A*26 and -*B locus haplotype analysis in the Japanese population

2 loci retrieved haplotype frequencies of HLA-A*26 and -*B are shown in Table 3. The haplotype frequency of HLA-A*26-B*51 was 3.2% in BD cases and 0.7% in controls. The difference of the frequencies was statistically significant and the OR was 4.64 (95% CI: 2.98–7.24). Within the HLA-B*51 negative subsets, the entire sets of haplotypes retrieving HLA-A*26 and -*B alleles positively associated with BD susceptibility (OR = 1.59, 95% CI: 1.33–1.90). Within these various haplotypes, the haplotype frequency of HLA-A*26-B*40 was 5.9% in BD cases and 3.9% in controls, and the OR was 1.68 (95% CI: 1.27–2.22). The haplotypes of HLA-A*26-B*39, and A*26-B*55 showed relatively high ORs, 10.97 (95% CI:4.12–29.22) and 15.12 (95% CI: 2.40–95.14), respectively, but these haplotypes were rare both in BD cases and controls in Japanese population.

**Table 3:** Haplotypes of HLA-A*26 and HLA-*B allele in the Japanese population.

Haplotype	Haplotype Frequency		*p*	*P*c*	OR (95% CI)
	BD cases (N = 611)	Controls (N = 2,955)			
HLA-A*26:HLA-B*51	3.20%	0.71%	1.13E-13	1.65E-11	4.64 (2.98–7.24)
HLA-A*26:non-HLA-B*51	15.32%	10.24%	3.31E-07	4.83E-05	1.59 (1.33–1.90)
A*26:B*07	0.39%	0.21%	0.23		1.90 (0.65–5.55)
A*26:B*13	0.33%	0.03%	0.00067	0.098	12.06 (1.93–75.52)
A*26:B*15	2.72%	2.05%	0.14		1.34 (0.90–1.98)
A*26:B*18	0.00%	0.02%	0.65		0.00 (−)
A*26:B*27	0.00%	0.02%	0.67		0.00 (−)
A*26:B*35	1.91%	1.95%	0.93		0.98 (0.62–1.54)
A*26:B*37	0.26%	0.00%	8.7E-05	0.013	—
A*26:B*38	0.00%	0.06%	0.40		0.00 (−)
A*26:B*39	1.09%	0.10%	1.9E-09	2.7E-07	10.97 (4.12–29.22)
A*26:B*40	5.91%	3.61%	0.00020	0.029	1.68 (1.27–2.22)
A*26:B*44	0.01%	0.07%	0.44		0.12 (0.00–65.61)
A*26:B*46	0.00%	0.18%	0.14		0.00 (−)
A*26:B*48	0.73%	0.27%	0.012	1.00	2.76 (1.20–6.34)
A*26:B*52	0.70%	0.27%	0.019	1.00	2.63 (1.14–6.08)
A*26:B*54	0.34%	0.29%	0.76		1.18 (0.40–3.49)
A*26:B*55	0.39%	0.03%	0.00011	0.016	15.12 (2.40–95.14)
A*26:B*56	0.00%	0.71%	0.0034	0.50	0.00 (-)
A*26:B*59	0.16%	0.00%	0.0025	0.37	—
A*26:B*61	0.32%	0.00%	1.5E-05	0.0022	—
A*26:B*67	0.05%	0.42%	0.0499	1.00	0.12 (0.01–1.53)

*The obtained *p* values were corrected for multiple testing using the Bonferroni method based on the number (n = 146) of haplotypes observed in the Japanese population of the current study. If the corrected *P (Pc)* value was greater than 1, it was set to 1.

### Literature Search and Meta-analysis

**Figure 1:**
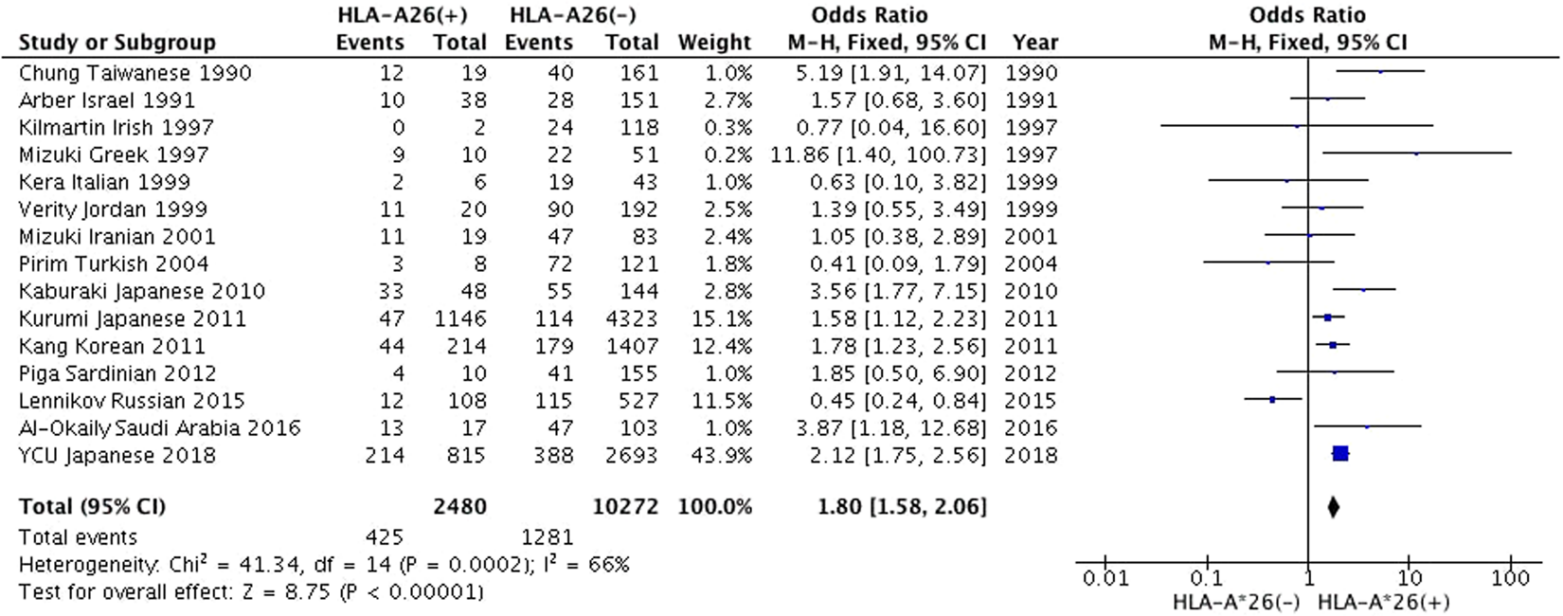
Forest plot from the meta-analysis on the association of HLA-A*26 and Behçet’s disease. Results are shown as OR, represented as a rectangle in the graph (size is proportional to the respective amount of data). The 95% CI are represented by bars.

**Figure 2:**
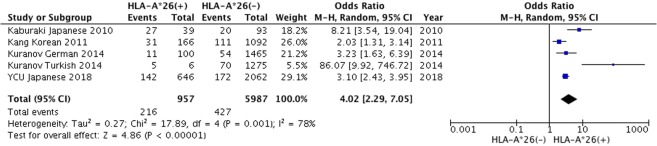
Forest plot from the meta-analysis on the association of HLA-A*26 and Behçet’s disease in the HLA-B*51-negative populations.

275 studies were identified through electronic search. 2 studies were added by manual database search. 85 studies were excluded after duplicate publications check. Full-texts of 99 studies were reviewed, and the final study included 14 independent case-control studies with 13 ethnicities, 1,104 BD patients and 8,140 healthy controls (Supplementary Fig. S1). Assessment of risk of bias for included studies was done through Newcastle-Ottawa Scale, and all included studies were of high quality with scores ranging from 7 to 9 (Table 4). Publication bias was assessed using a funnel plot (Supplementary Fig. S2). A synthesized analysis of data from a number of publications showed that the OR of HLA-A*26 among BD patients was 1.62 (95% CI: 1.09–2.39). Combined with our genotyping data, the modified OR became 1.80 (95% CI: 1.58–2.06). (Fig. 1). Additionally, within the HLA-B*51-negative populations, the entire OR combined with our genotyping data became 4.02 (95% CI: 2.29–7.05) (Fig. 2).

**Table 4:** Characteristics of the studies included for meta-analysis.

Author	Year	Country	Publication language	Cases	Controls	OR	95% CI	Selection of Controls	genotyping methods	NOS score
Chung *et al*.	1990	Taiwan	Chinese	12/52	7/128	5.19	1.91–14.07	N/A	MLCT + FC	7
Arber *et al*.	1991	Israel	English	10/38	28/151	1.57	0.68–3.60	N/A	MLCT + FC	7
Kilmartin *et al*.	1997	Ireland	English	0/24	2/96	0.77	0.04–16.60	CC	MLCT + FC	8
Mizuki *et al*.	1997	Greece	English	9/31	1/30	11.86	1.40–100.73	CC	MLCT + FC	8
Kera *et al*.	1999	Italy	English	2/21	4/28	0.63	0.10–3.82	CC	MLCT + FC	8
Verity *et al*.	1999	Jordan, Palestine	English	11/101	9/111	1.39	0.55–3.49	HC	PCR-SSP	8
Mizuki *et al*.	2001	Iran	English	11/58	8/44	1.05	0.38–2.89	CC	PCR-SSP	9
Pirim *et al*.	2004	Turkey	English	3/75	5/54	0.41	0.09–1.79	CC	PCR-SSP	9
Kaburaki *et al*.	2010	Japan	English	33/88	15/104	3.56	1.77–7.15	CC	PCR-SSO	8
Kang *et al*.	2011	Korea	English	44/223	170/1,398	1.78	1.23–2.56	CC	PCR-SSO	9
Kurumi *et al*.	2011	Japan	Japanese	47/161	1099/5308	1.58	1.12–2.23	CC	MLCT + FC, PCR-rSSO	9
Piga *et al*.	2012	Sardinia	English	4/45	6/120	1.85	0.50–6.90	CC	PCR-SSP	9
Lennikov *et al*.	2015	Russia	English	12/127	96/508	0.45	0.24–0.84	CC	MLCT + FC	7
Al-Okaily *et al*.	2016	Saudi Arabia	English	13/60	4/60	3.87	1.18–12.68	CC	PCR-SSO	9

In the columns titled cases and controls, n/N refers to n: number of HLA-A*26 (+) participants; N: total number of participants. In the Selection of Control column; CC: community controls; HC: hospital controls. In the column titled genotyping method, MLCT + FC: microlymphocytotoxicity method and flow cytometry; PCR-SSO; polymerase chain reaction-sequence specific oligonucleotide; rSSO: reverse sequence specific oligonucleotide; SSP: sequence specific primers.

### Regional disparities in the contribution of HLA-A*26 to the onset of BD

**Figure 3:**
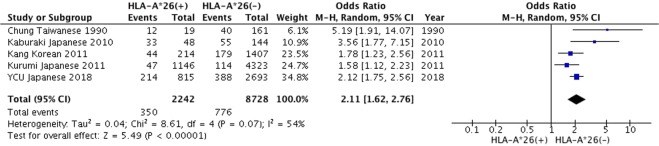
Subgroup meta-analysis by geographical region of the relationship between HLA-A*26 and Behçet’s disease in Northeast Asia.

**Figure 4:**
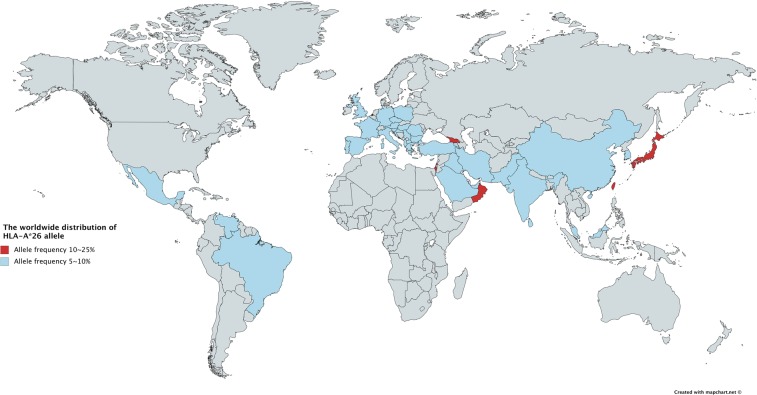
The worldwide distribution of HLA-A*26. The frequency of HLA-A*26 in regions and countries colored in red and blue are of 10~25%, and 5~10%, respectively. As for the other regions, the frequency was below 5%. The figure, made using mapchart.net, is licensed under CC BY-SA 4.0 license (https://creativecommons.org/licenses/by-sa/4.0/).

Subgroup analysis suggested that the association of HLA-A*26 between the onset of BD was statistically significant in the Northeast of Asia (OR = 2.11, 95% CI: 1.62–2.76) (Fig. 3), but not in the Middle East (OR = 1.39, 95% CI: 0.79–2.45), or in Europe (OR = 1.85, 95% CI: 0.55–6.22) (Supplementary Figs S3 and S4, respectively). The worldwide distribution of HLA-A*26 is shown in Fig. 4^13^.

## Discussion

Despite its worldwide presence, BD has a much higher prevalence in countries along the ancient Silk Route that extends from the Mediterranean basin to the far Eastern Asia. Turkey has the highest prevalence of BD in the world, with 20–420 cases per 100,000 reported^14–17^. Compared to that, the prevalence rates range from 7.3–30.5 cases per 100,000 in Korea, China, Iran, Saudi Arabia, and Japan^18^. Strong genetic association between HLA-B*51 and BD has been identified in numerous ethnicities and counties^3,18^. We have previously reported that HLA-A*26 was significantly and independently associated with the risk of BD, apart from HLA-B*51 in the Japanese population^12^. Several studies including various ethnic groups and populations indicated both positive and negative associations between HLA-A*26 and BD^4–8,11,12,19–27^. In our current study, we aimed to summarize how HLA-A*26 is involved in the risk of BD within various geographical regions and ethnicities. In our current genotyping study, within a total of 611 BD patients, almost half of the patients did not carry HLA-B*51 alleles, and within these HLA-B*51-negative populations, almost half of the patients carried HLA-A*26. This indicated that almost a quarter of the total BD patients were independently influenced by HLA-A*26 in the Japanese population. Moreover, the OR of HLA-A*26 to BD was 2.12, and was as high as 3.10 within the HLA-B*51 negative subsets. As shown in Table 3, the haplotype frequencies retrieving HLA-A*26 and -*B locus indicated several supporting findings. According to the pooled database of *HLA* haplotypes in Japanese population, which include 8,138 families and 31,665 individuals, the haplotype frequency of HLA-A*26:01-B*51:01 was 0.46% and LD value was −0.219 and RD value was −0.324 (http://hla.or.jp, accessed December 2018). That indicated HLA-A*26:01 and B*51:01, the predominant suballeles in both BD patients and controls, were not in the linkage disequilibrium, which supported to re-confirm HLA-A*26 was an independent genetic risk factor apart from HLA-B*51. Within the HLA-B*51 negative subsets, some haplotypes retrieving HLA-A*26 and non- HLA-B*51 alleles showed positive association with susceptibility to BD, i.e., HLA-A*26 -B *39, *40, and *55. Of note, none of these -*B alleles showed significant independent risk association with BD in our current study. This may suggest the existence of other latent genetic risk alleles between HLA-A*26 and these -*B loci, for instance, possible involvement of retrieving HLA-*C, and -*E alleles^28^. Further studies will be necessary to clarify the possible involvement of these alleles to the susceptibility of BD.

As reported by Hughes, *et al*., no association was found between HLA-A*26 and BD in their larger number case-control study within the Turkish population. They concluded the lack of association owes to the low frequency of HLA-A*26 in the Turkish population^29^. On the other hand, Ombrello, *et al*. reported that the HLA-A*26 allele independently influenced the risk of BD apart from HLA-B*51 within another Turkish population, according to the HLA imputation analysis of their pooled GWAS results. They also clarified that five amino acid residues of HLA-B and two residues of HLA-A were significantly associated with BD. Id est, positions 97, 116, 152, and 67 of HLA-B and positions 161 and 97 of HLA-A have protective or risk effects to BD. Their studies suggested Arg 97 of HLA-A was significantly and independently associated with the risk of BD onset (OR = 1.3, 95% CI:1.1–1.4)^26^. Residue 97 of the HLA-A molecule is a component of the pocket F, located in the antigen-binding groove, and interact with the C-terminus of the presented peptide. Residue position 2 and the C-terminus of the presented peptide are called main anchor positions which define the binding affinity and specificity of HLA class I molecules^30^. HLA-A*26:01 has Arg at position 97 and that corresponds to the present risk residue mentioned above. HLA-B*51 has a Bw4 epitope in the *α*1-binding pocket which interact with the killer immunoglobulin-like receptors (KIR) 3DL1 and 3DS1 to regulate the activities of natural killer (NK) cells and a subset of cytotoxic T lymphocytes (CTLs)^31^. Their group also suggested a potential role of activating KIR3DS1 alleles in BD patients with ocular manifestations independent of HLA-B*51^32^. Though HLA-A*26 has a strong linkage with ocular manifestations in the Northeast Asian population, as far as KIR interaction is concerned, HLA-A*26 is not a direct ligand of KIR molecules. In another words, HLA-A*26 does not have an epitope which could be directly recognized by KIR to regulate the activation of NK cells or CTLs. That may suggest a different pathophysiology from HLA-B*51 related KIR interaction underlies the development of BD in the case of HLA-A*26 triggered KIR interaction pathways. Further studies are required to ascertain the hypothesis suggested above.

The worldwide distribution of HLA-A*26 is unique, as it is especially frequent in Northeast Asia, Oman, Georgia, and in the Israeli Jewish population (Fig. 4)^13^. In Northeast Asia, HLA-A*26 is more commonly found in the Western Pacific Rim, i.e. Taiwan, Ryukyu (Okinawa islands), and the Japan Islands. In our subgroup analysis arranged by geographical areas, positive association between HLA-A*26 and BD was found in Northeast Asia, but not in the Middle East or in Europe. This may owe to the higher distribution of this allele in the Northeast Asian region, especially in the Western Pacific Rim, we might be able to find the association between HLA-A*26 and BD in these areas more apparently.

In spite of the relatively high prevalence of HLA-A*26 in the Jewish population in Israel, no positive association between HLA-A*26 and BD was reported^25^. We believe this is due to the high heterogeneity of the Israeli Jewish population: A large proportion of them is of Ashkenazi Jewish origin, of which 21.7% were HLA-A*26 positive^33^. However, as reported in the previous papers, most of the Jewish BD patients were of non-Ashkenazi origin^25,34^. The lack of BD/HLA-A*26 association in this particular region might owe to the low frequency of HLA-A*26 in the non-Ashkenazi Jewish population. In addition, results of studies concerning the *HLA* genotyping of BD patients in Georgia and Oman were not found during our research.

In their GWAS results, Abi-Rached, *et al*. reported that between all three Neanderthals found in the Vindija Cave, northern Croatia, had the HLA-A*02, C*07:02, and C*16 alleles. Moreover, the pooling of these three Neanderthals sequence infers their possession of HLA-B*07, -B*51, and either HLA-A*26 or its close relative A*66^35^. It was suggested that the presence of HLA-B*51 in Eurasians, together with B*07, C*07:02, C*16:02, might be the result of admixture with the Neanderthals, which occurred after out-of-Africa migration until 40–30,000 years ago^35,36^. It is believed that the adaptive introgression of the Neandertal alleles has significantly involved in the construction of the modern humans’ immune systems and contributed to mediate the host defense immune mechanism against lethal infectious agents which the ancestors of modern humans newly encountered in the frontiers of the Eurasian continent during the period of the great human expansion. It is of interest that both HLA-B*51^37^ and HLA-A*26 alleles, which were identified as risk alleles of BD, might have been introgressed from the archaic human species, Neanderthals. Further studies will be needed for the better understanding of these mysterious relationships between HLA-B*51, HLA-A*26, Neanderthal alleles and BD. It is also worth noting that modern human’s ancestors lived in an environment where infectious diseases were mostly endemic, and under the influence of endemic environmental agents, infective microbial organisms led to genetic selection in order to produce more effective pro-inflammatory response to encourage the resistance to specific infections. However, these effective and high-potency immune systems could lead to immune-mediated inflammatory disease as an undesirable adverse effect^38^. *Yersinia Pestis*, the cause of Plague, is reported to have evolved near China, 20–15,000 years ago^39^. *Yersinia Pestis* has been a lethal infectious agent to human beings, and several studies have suggested HLA-B*51:01 had a protective role in the host response against the *Yersinia Pestis* infection^40^. It is assumed that the bottleneck effect following the high mortality rate of plague epidemics might have led the expansion of HLA-B*51:01 associated increased pro-inflammatory phenotypes and reservation of this complex genetically determined trait^40^. In other words, HLA-B*51 associated BD is suspected to be a secondary and undesirable side effect of the immunological advantages rendered by HLA-B*51 in activating NK cells and CTLs in response to these lethal infections^37^. Currently, we do not have enough knowledge on how HLA-A*26 contributed to protect modern human ancestors from life-threatening infections in the human immune history, we believe comprehensive investigations and better understanding of HLA-A*26 will lead us to a better understanding of BD pathogenesis.

In conclusion, we have performed the genotyping of Japanese BD patients and confirmed that HLA-A*26 was the susceptibility allele for BD in the Japanese population. Especially in the HLA-B*51-negative BD populations, HLA-A*26 was significantly associated with the onset of BD. A combination of our genotyping data with other data extracted from publications showed the association of BD and HLA-A*26 was geographically significant in Northeast Asia, but not in the Middle East or in Europe.

## Methods

### BD patients and controls

611 Japanese BD patients and 2,955 unrelated ethnically matched healthy controls were enrolled in this study. The diagnosis of BD was established according to standard criteria^41^ proposed by the Japan Behçet’s disease Research Committee. All procedures, data collection, and handling were performed according to the principles of the Good Clinical Practice and Declaration of Helsinki. This study was approved by the Research Ethics Committee of the Medical Faculty, Yokohama City University. The study details were explained to all participants before obtaining the informed consent for genetic screening. Blood samples were collected after study participants agreed and signed informed written consent. Banked and de-identified samples were used for this study.

### HLA genotyping

We genotyped *HLA-A* and *HLA-B* alleles for 611 cases and 737 controls with Luminex reverse sequence-specific oligonucleotides and bead kits (One Lambda). For the remaining 2,218 controls, we performed an imputation analysis of *HLA-A* and *HLA-B* with our GWAS data using SNP2HLA^42^ and a reference panel of 530 pan-Asian samples^43^. The χ^2^ test was used to analyze categorical variables.

### Literature Search and Meta-analysis

Meta-analysis was performed through the method proposed by the Preferred Reporting Items for Systematic Reviews and Meta-Analysis (PRISMA)^44^, using the Review Manager software (version 5.3) for statistical analysis. This protocol has been registered in the international prospective register of systematic reviews (PROSPERO) as number CRD42017073887. Relevant studies were identified using the PubMed/Medline, Embase, Web of Science, CENTRAL database and through manual literature search in December 2018; no language restriction was used for published studies. Studies fulfilling the following inclusion criteria were included in the meta-analysis: (1) case-control studies; (2) studies reporting an association between *HLA* and BD; (3) genetic association studies; and (4) independent studies without repeat reports on the same populations or subpopulations. In this study, we performed the quantitative synthesis of the extracted data which contain the results of phenotype frequency, hence extracted data which contain only allele frequency results without phenotype frequency results were included in qualitative synthesis, but not into the quantitative synthesis. Two of the authors (JN and GI) individually assessed the bias of included-studies using the Newcastle-Ottawa Scale^45^. Pooled ORs and the corresponding 95% CIs were synthesized with the random-effects model. Heterogeneity was assessed using the I^2^ statistic.

We also performed subgroup analysis to identify the possible underlying heterogeneity according to ethnic and geographic (Northeast Asia, Middle East, and Europe) repartitions of the BD patients in the studies. Publication bias was assessed using a funnel plot of the Review Manager software.

## Supplementary information

Supplemental Materials
