# Supplementary material for: The association analysis between HLA-A*26 and Behçet’s disease
Source: Sci Rep. 2019 Mar 14;9:4426. doi: 10.1038/s41598-019-40824-y (PMC6418292; doi:10.1038/s41598-019-40824-y)
Supplement: Supplementary file 1 — Supplemental Materials [file 41598_2019_40824_MOESM1_ESM.pdf]

Supplemental Materials

The association analysis between *HLA-A*26* and Behçet's disease

Jutaro Nakamura*[1, 2], Akira Meguro[1], Genji Ishii[3], Takahiro Mihara[4], Masaki Takeuchi[1], Yuki Mizuki[1], Kentaro Yuda[1], Takahiro Yamane[1], Tatsukata Kawagoe[1], Masao Ota[1,5], and Nobuhisa Mizuki[1]

[1]Department of Ophthalmology and Visual Science, Yokohama City University Graduate School of Medicine, Yokohama, Japan
[2]Department of Ophthalmology, Heisei Yokohama Hospital, Yokohama, Japan
[3]Product Strategy Department, PFU Limited, a Fujitsu Company, Yokohama, Japan
[4]Department of Anesthesiology and Critical Care Medicine, Yokohama City University Graduate School of Medicine, Yokohama, Japan
[5]Department of Medicine, Division of Gastroenterology and Hepatology, Shinshu University School of Medicine, Matsumoto, Japan

***Corresponding Author**
Jutaro Nakamura, M.D. & Ph.D., Department of Ophthalmology and Visual Science, Yokohama City University Graduate School of Medicine, 3-9 Fukuura, Kanazawa, Yokohama, Kanagawa, 236-0004, Japan

TEL +81-45-860-1777
FAX +81-45-860-1745
E-mail: nakamura.jutaro@hmw.gr.jp

Supplementary Figure S1. The PRISMA flow diagram of the study selection and exclusion (Moher, Liberati, Tetzlaff, & Altman, 2009).

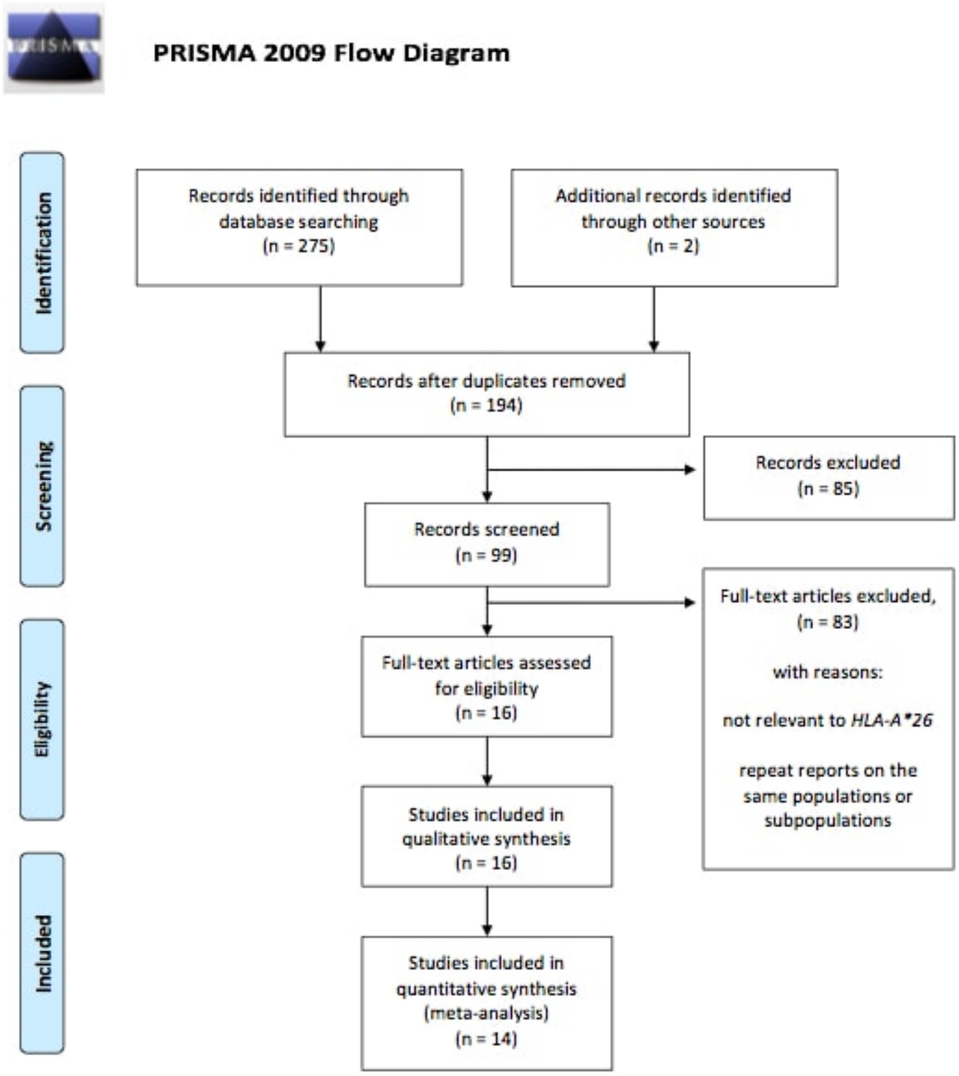

Note: After PRISMA flow diagram (https://doi.org/10.1371/journal.pmed.1000097)[1].

Supplementary Figure S2. Funnel plots of the included studies
SE: standard error, OR: odds ratio

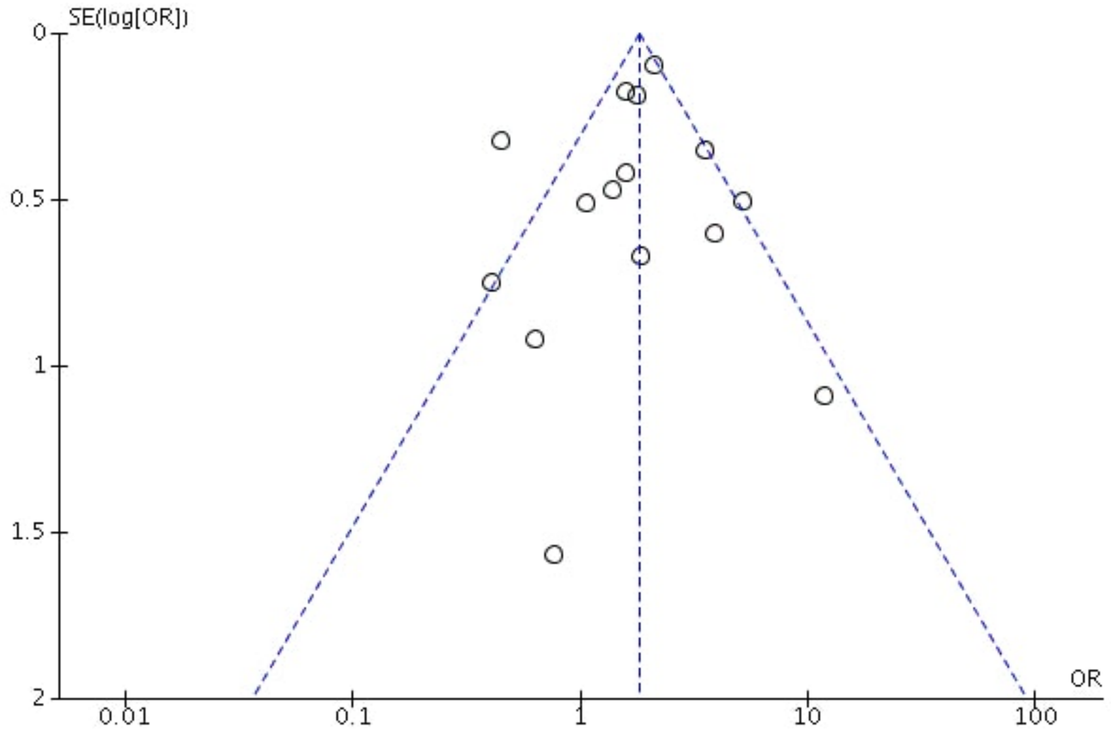

Supplementary Figure S3. Subgroup meta-analysis arranged by geographical region of the relationship between *HLA-A*26* and Behçet's disease in the Middle East

Study or Subgroup	HLA-A*26(+) Events	HLA-A*26(+) Total	HLA-A*26(+) Events	HLA-A*26(+) Total	Weight	Odds Ratio M-H, Random, 95% CI	Year
Arber Israel 1991	10	38	28	151	26.8%	1.57 [0.68, 3.60]	1991
Verity Jordan 1999	11	20	90	192	23.5%	1.39 [0.55, 3.49]	1999
Mizuki Iranian 2001	11	19	47	83	21.0%	1.05 [0.38, 2.89]	2001
Pirim Turkish 2004	3	8	72	121	11.9%	0.41 [0.09, 1.79]	2004
Al-Okaily Saudi Arabia 2016	13	17	47	103	16.8%	3.87 [1.18, 12.68]	2016
**Total (95% CI)**		**102**		**650**	**100.0%**	**1.39 [0.79, 2.45]**	
Total events	48		284				

Heterogeneity: Tau² = 0.13; Chi² = 5.88, df = 4 (P = 0.21); I² = 32%
Test for overall effect: Z = 1.13 (P = 0.26)

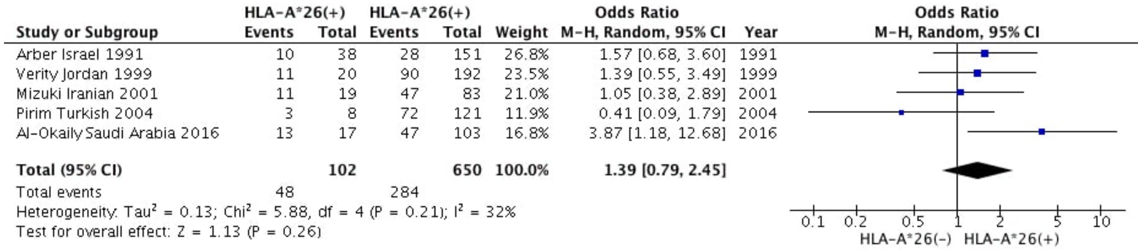

Supplementary Figure S4. Subgroup meta-analysis arranged by geographical region of the relationship between *HLA-A*26* and Behçet's disease in Europe

Study or Subgroup	HLA-A*26(+) Events	HLA-A*26(+) Total	HLA-A*26(-) Events	HLA-A*26(-) Total	Weight	Odds Ratio M-H, Random, 95% CI	Year
Kilmartin Irish 1997	0	2	24	118	12.7%	0.77 [0.04, 16.60]	1997
Mizuki Greek 1997	9	10	22	51	21.9%	11.86 [1.40, 100.73]	1997
Kera Italian 1999	2	6	19	43	27.3%	0.63 [0.10, 3.82]	1999
Piga Sardinian 2012	4	10	41	155	38.1%	1.85 [0.50, 6.90]	2012
**Total (95% CI)**		**28**		**367**	**100.0%**	**1.85 [0.55, 6.22]**	
Total events	15		106				

Heterogeneity: Tau² = 0.55; Chi² = 4.71, df = 3 (P = 0.19); I² = 36%
Test for overall effect: Z = 1.00 (P = 0.32)

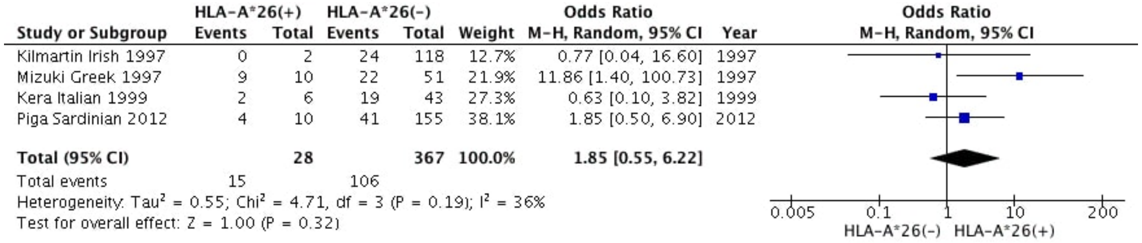

Reference:

1 Moher, D., Liberati, A., Tetzlaff, J., Altman, D. G. & The, P. G. Preferred Reporting Items for Systematic Reviews and Meta-Analyses: The PRISMA Statement. *PLOS Medicine* **6**, e1000097, doi:10.1371/journal.pmed.1000097 (2009).
